# Prenatal Depression and Its Associated Risk Factors Among Pregnant Women in Bangalore: A Hospital Based Prevalence Study

**DOI:** 10.3389/fpubh.2019.00108

**Published:** 2019-05-03

**Authors:** B. Sheeba, Anita Nath, Chandra S. Metgud, Murali Krishna, Shubhashree Venkatesh, J. Vindhya, Gudlavalleti Venkata Satyanarayana Murthy

**Affiliations:** ^1^Research Assistant, Indian Institute of Public Health Hyderabad, Public Health of Foundation of India, Bangalore, India; ^2^Wellcome Trust DBT India Alliance, Intermediate Fellow in Clinical and Public Health, Indian Institute of Public Health Hyderabad, Public Health Foundation of India, Hyderabad, India; ^3^Professor, Department of Community Medicine, Pt. Jawahar Lal Nehru Memorial Medical College, Belgavi, India; ^4^Consultant, FRAME, Mysore, India; ^5^Indian Institute of Public Health Hyderabad, Public Health Foundation of India, Hyderabad, India

**Keywords:** prenatal depression, pregnant women, domestic violence, marital discord, social support, pregnancy related anxiety, Banglaore

## Abstract

**Background:** Depression is the commonest psychological problem that affects a woman during her perinatal period worldwide. The risk of prenatal depression increases as the pregnancy progresses and clinically significant depressive symptoms are common in the mid and late trimester. There is a paucity of research on depression during the prenatal period in India. Given this background, the present study aimed to assess the prevalence of prenatal depression and its associated risk factors among pregnant women in Bangalore, Southern India.

**Methods:** The study was nested within an on-going cohort study. The study participants included 280 pregnant women who were attending the antenatal clinic at Jaya Nagar General Hospital (Sanjay Gandhi Hospital) in Bangalore. The data was collected by using a structured questionnaire which included. Edinburgh Postnatal Depression Scale (EPDS) to screen for prenatal depression.

**Results:** The proportion of respondents who screened positive for prenatal depression was 35.7%. Presence of domestic violence was found to impose a five times higher and highly significant risk of developing prenatal depression among the respondents. Pregnancy related anxiety and a recent history of catastrophic events were also found to be a positive predictors of prenatal depression.

**Conclusion:** The high prevalence of prenatal depression in the present study is suggestive of its significance as a public health problem. Health care plans therefore can include screening and diagnosis of prenatal depression in the antenatal care along with other health care facilities provided.

## Introduction

The relationship between a pregnant woman and her developing fetus is possibly the most earnest and overwhelming but perplexing of all human relationships. Pregnancy entails physiological, hormonal and psychological changes which could increase the probability of mental and emotional changes resulting in depression, anxiety or psychological distress in the pregnant mother (1).

Maternal and Child Health Programmes in developing countries are commonly focused upon improving the nutritional status and less importance is given toward a woman's emotional and mental health during and after pregnancy (2, 3). Poor mental health of the woman during pregnancy could have profound consequences for the mother and her child in terms of adverse pregnancy outcomes and offspring development (4–7). Most of the existing data, research, and practice policies with regard to perinatal mental disorders center on the postnatal period and there is less research related depression during pregnancy (8, 9).

Depression is the most common psychological problem that affects a woman worldwide during the perinatal period (3, 10). About 15 % of women are known to be depressed at some point during their lifetime and more predominantly during pregnancy and after childbirth (11). The risk of prenatal depression increases significantly as the pregnancy progresses and clinically significant depressive symptoms are common in the mid and late trimester (12). The prevalence rates of prenatal depression differ between high, middle and low—income countries. Studies from various countries around the world show a prevalence rate ranging from as low as 4% to as high as 81% (13–16). The prevalence rate is reported to be lower in high income countries like Australia 7% (17), Hong Kong 4.4% (18), Finland 7.7% (19), and higher in many of the low-income countries like Pakistan 64.6% (20), Bangladesh 18% (13), Nigeria 24.5% (14), and Ethiopia 24.94% (15). The prevalence of depression in India is varies from 9.18% in one study to 36.7 % reported in another study (21, 22).

Even though prenatal depression is an important public health problem, most studies related to maternal depression are focused on post-natal depression and its outcomes; hence there is paucity of research on depression during the prenatal period, especially from India (8). The importance of screening for depression during pregnancy is that prenatal depression, if not treated and diagnosed early, may continue as postnatal depression (23–25) later on and could also result in an adverse influence on birth outcomes and offspring development. Given this background, the present study aimed to assess the prevalence of prenatal depression and its associated risk factors among pregnant women in Bangalore, Southern India.

## Materials and Methods

### Study Setting and Participants

The study sample included of pregnant women who were attending the antenatal clinic at Jaya Nagar General Hospital (also known as Sanjay Gandhi Hospital), which is a public sector hospital in Bangalore. The study was nested within an ongoing cohort study, the study protocol of which was published earlier (26). The eligibility criteria included women above or equal to 18 years of age, with confirmed pregnancy of < 6 months (< 24 weeks) and having no obstetric or medical complication in the present pregnancy. The study analyzed the data of 280 pregnant women who had enrolled and completed the baseline visit for the study between August 2017 and April 2018.

### Data Collection

Data was obtained from the pregnant women by means of an interview, after obtaining written informed consent. A participant information sheet that explained the purpose and nature of the study was issued to those who were willing to participate in the study. The respondents were ensured about privacy and confidentiality of data. The interview process employed the use of a structured questionnaire installed in an Android tablet App. The App included questions about socio-demographic data, obstetric history, medical history and measures for depression, social support, marital discord, domestic violence, and pregnancy related anxiety described below. Data related history of any mental illness and recent catastrophic event was also recorded. Calibrated instruments were used to measure height and weight and calculate the Body Mass Index (BMI). Data on hemoglobin estimation was obtained from hospital records. Depression, being the outcome variable was measured using Edinburg Postnatal Depression Scale (EPDS).

### Study Measures

#### Depression

EPDS is a widely used 10-item self-reporting instrument, specifically designed for assessing both prenatal as well as postnatal depression. It has a sensitivity of 86%, specificity of 78% and positive predictive value of 73% (27). EPDS has been validated for detecting depression in both antepartum and postpartum mothers in many countries. This scale consists of 10 short questions with a choice of four answers that closely reflects about how she was feeling over the past 7 days. Scores are recorded as 0, 1, 2, and 3 according symptom severity. Certain question items (i.e., 3, 2, 1, and 0) are scored in a reverse manner. Respondents who score 13 and above are likely to be suffering from depression and should seek medical attention.

#### Social Support

The Multidimensional Scale of Perceived Social Support Scale (MSPSS) used to measure social support includes 12 questions, and is validated for use in the South Asian population (28, 29). These questions directly address the adequacy of social support and have a 7-point rating scale ranging from “very strongly disagree” to “very strongly agree.” The scale assesses the perceptions of social support adequacy from three specific sources: family, friends, and “significant other.” A score of < 2 is considered as low support, a score of 3–5 as moderate support while score of more than five indicates high support.

#### Marital Discord

The Revised Dyadic Adjustment Scale (30, 31) measures seven dimensions of relationship among partners within three categories: decision making, values and affection. It consists of 14 items in which the respondents can rate their relationship on a 6-point scale. Scores range from 0 to 69; higher the score greater, is the relationship and *vice versa*. The cut- off score was taken as 48.

#### Spouse Physical and Sexual Violence

Spouse physical and sexual violence was measured using the Modified Conflict Tactics Scale (32). It is effective and useful in measuring domestic violence in diverse cultural settings. The scale has 9 questions wherein the respondents affirm whether domestic violence was present or absent.

#### Socio Economic Scale

The socio-economic class of the respondents was measured by the Modified Kuppuswamy Socio Economic Scale (33). The scale uses education, occupation of the head of the family and monthly family income to calculate socio-economic status. The scores awarded to education and occupation of the head of the family remains unchanged. Revised Consumer Price Index–IW (industrial workers) is used to calculate the monthly income range. The socio-economic status is classified as upper class, upper middle class, lower middle class, upper lower class and lower class.

#### Pregnancy Related Anxiety

The 10-item Pregnancy Related Anxiety Questionnaire (PRAQ) was used to screen for pregnancy anxiety (34). It appears to have good psychometric and predictive validity for child-birth and childhood outcomes. Each item is scored on a 4-point scale with cut-off scores of 28 and 24 for nulliparous and multiparous women and the internal consistency (Cronbach's alpha) of PRAQ was seen to be 0.79. A score of more than 28 was considered as anxious.

### Statistical Analysis

Data were retrieved from the data server. This was followed by data cleaning and analysis using SPSS version 22. Descriptive statistics such as percentage, means and standard deviation were used to summarize the socio demographic data. An EPDS score of 13 and above pointed toward the likelihood of presence of depression. The independent variables were categorized to analyze the association between each independent and outcome variable using a bivariate analysis to calculate the Crude Odd's Ratio with 95% Confidence Interval. Those variables that were associated at a *P*-value of < 0.2 in the bivariate analysis were entered into a multivariate logistic regression model to calculate the Adjusted Odd's Ratio and to eliminate the effects of confounding. Variables with a *P*-value of < 0.05 in the multivariate analysis were considered to be significant.

### Ethical Considerations

The study was approved by the Ethical Committee of Indian Institute of Public Health Bangalore campus (IIPHHB/TRCIEC/118/2017). Written informed consent was obtained from the pregnant mothers and they were assured of confidentiality and privacy of records.

## Results

### Socio Demographic Characteristics of the Respondents

Table 1 shows the frequency distribution of socio demographic characteristics of the respondents. Of the 280 pregnant mothers, majority (72.9%) of them belonged to the age group of more than 20 years, the mean age of the respondents being 23.02 ± 3.40 years. Over two-thirds among them (72.1%) were Muslim and 40.4% had completed High school. While 92.1% were housewives, the spouses of over half of the respondents (51.8%) were semi- skilled workers. According to the Kuppuswamy Socio economic status scale, more than half of the respondents (57.5%) belonged to Upper Lower class. Nearly seventy percent of the pregnant mothers had no blood relationship with their husbands, where as a notable 13.2% said that their husbands were a first cousin from their mother's side.

**Table 1:** Socio demographic characteristics of the study participants (*N* = 280).

**Socio demographic characteristics**	**Frequency (*n* = 280)**	**Percentage (%)**
**AGE GROUP (IN YEARS)**		
≤ 20	76	27.1
>20	204	72.9
**RELIGION**		
Hinduism	73	26.1
Christianity	05	1.8
Islam	202	72.1
**EDUCATIONAL QUALIFICATION OF THE RESPONDENTS**		
Illiterate	07	2.5
Primary school	08	2.9
Middle school	77	27.5
High school	113	40.4
PUC or diploma	52	18.6
Graduate	23	8.2
**EDUCATIONAL QUALIFICATION OF THE HUSBANDS**		
Illiterate	36	12.9
Primary school	20	7.1
Middle school	71	25.4
High school	95	33.9
PUC or diploma	36	12.9
Graduate and post-graduate	22	7.8
**OCCUPATION OF THE RESPONDENTS**		
Unskilled worker	11	3.9
Semi-skilled worker	10	3.6
Clerical or farmer	01	0.4
Housewife	258	92.1
**OCCUPATION OF THE HUSBANDS**		
Unemployed	01	0.4
Unskilled worker	96	34.3
Semi-skilled worker	145	51.8
Skilled worker	35	12.5
Clerical or farmer	01	0.4
Semi professional	02	0.7
**SOCIO ECONOMIC STATUS**		
Upper middle class	36	12.9
Lower middle class	83	29.6
Upper Lower class	161	57.5

### Prevalence and Magnitude of Prenatal Depression Among the Pregnant Women

**Figure 1:**
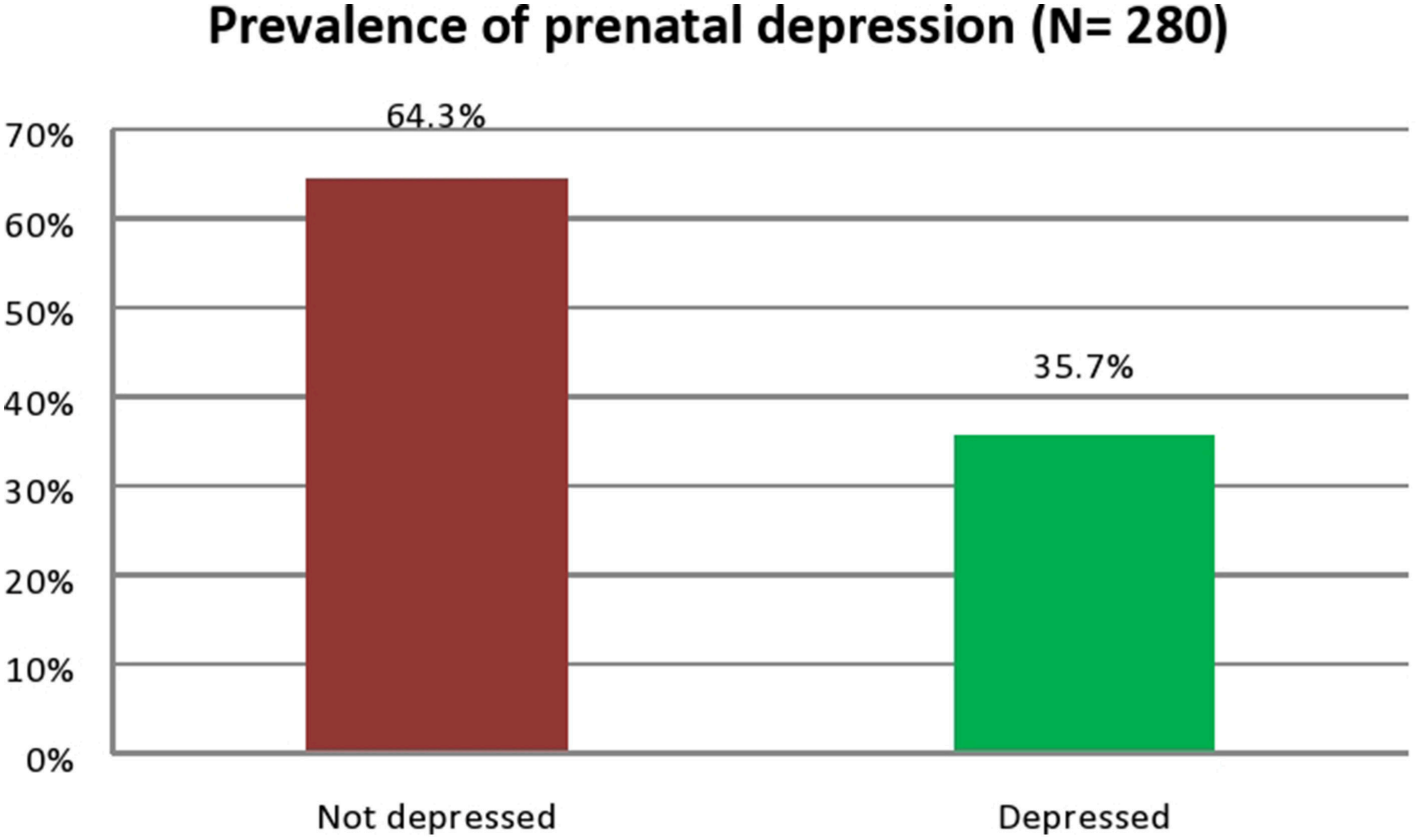
Prevalence of prenatal depression among the pregnant women (*N* = 280).

Of the 280 pregnant mothers, the proportion of those who screened positive for prenatal depression was 35.7% (100) suggesting a high probability of clinical depression (Figure 1). The mean EPDS score among the respondents was 10.61± 7.48.

### Association of Prenatal Depression With Socio-Demographic Characteristics

The association of socio-demographic factors like age group, educational qualification, occupation, and socio-economic status of the respondents with depression was non-significant on bivariate analysis (*p*-value >0.05). This is seen from Table 2.

**Table 2:** Association of socio demographic characteristics with depression among the pregnant women (*N* = 280).

**Socio demographic Characteristics**	**Depressed mothers (>13) (*n* = 100)**	**Non-depressed mothers (<13) (*n* = 180)**	**Bivariate analysis-crude odd's ratio (95% CI)**	***P*-value**
**AGE GROUP (IN YEARS)**				
≤ 20	29 (29%)	47 (26.1%)	1.156 (0.670–1.994)	0.603
>20	71 (71%)	133 (73.9%)	1	
**EDUCATIONAL QUALIFICATION OF THE RESPONDENTS**				
< High school	77 (77.0%)	126 (70.0%)	0.710 (0.403–1.250)	0.236
≥High school	23 (23.0%)	54 (30.0%)	1	
**EDUCATIONAL QUALIFICATION OF THE HUSBANDS**				
< High school	49 (49%)	78 (43.3%)	1.256 (0.769–2.052)	0.362
≥High school	51 (51%)	102 (56.7%)	1	
**OCCUPATION OF THE RESPONDENTS**				
Working	08 (8.0%)	15 (8.3%)	1.045 (0.427–2.559)	0.922
Housewife	92 (92.0%)	165 (91.7%)	1	
**OCCUPATION OF THE HUSBANDS**				
Skilled worker	12 (12%)	26 (14.4%)	1	
Semi/unskilled worker	88 (88%)	154 (85.6%)	1.238 (0.595–2.575)	0.568
**SOCIO ECONOMIC STATUS**				
Upper middle class	12 (12.0%)	24 (13.3%)	1	
Lower middle class	26 (26.0%)	57 (31.7%)	0.912 (0.396–2.100)	0.829
Upper lower class	62 (62.0%)	99 (55.0%)	1.253 (0.584–2.686)	0.563

### Association of Prenatal Depression With Obstetric History (Table 3)

On bivariate analysis, the number of pregnancies (gravida) and unplanned pregnancy showed an association with depression at a *P*-value of < 0.2. However, there was no significant association observed from multivariate logistic regression analysis.

**Table 3:** Association of depression with obstetric history of the pregnant women (*N* = 280).

**Obstetric history**	**Depressed mothers (>13) (*n* = 100)**	**Non-depressed mothers (<13) (*n* = 180)**	**Bivariate analysis crude OR (95% CI)**	***P*-value**	**Multivariate analysis adjusted OR (95% CI)**	***P*-value**
**GRAVIDITY**						
Primigravida	37 (37.0%)	81 (45.0%)	1		1	
Multigravida	63 (63.0%)	99 (55.0%)	1.393 (0.844–2.299)	0.195	1.386 (0.793–2.424)	0.252
**PARITY**						
Primipara	43 (43.0%)	86 (47.8%)	1			
Multipara	57 (57.0%)	94 (52.2%)	1.213 (0.741–1.984)	0.442		
**HISTORY OF ABORTION**						
Yes	25 (25.0%)	37 (20.6%)	0.776 (0.435–1.385)	0.391		
No	75 (75.0%)	143 (79.4%)	1			
**PREGNANCY UNPLANNED**						
Yes	52 (52.0%)	74 (41.1%)	1.552 (0.949–2.538)		1.604 (0.925–2.782)	
No	48 (48.0%)	106 (58.9%)	1	0.080	1	0.092

### Association Between Social Support, Marital Discord, Domestic Violence, Prenatal Anxiety, Consanguinity, and Catastrophic Events With Prenatal Depression (Table 4)

Association with low social support and presence of marital discord was significant on bivariate analysis but not in multivariate logistic regression. Presence of domestic violence was found to impose a five times higher and highly significant risk of developing prenatal depression among the respondents (COR = 5.438; 95% CI: 1.6–17.5, AOR = 5.916; 95% CI: 1.7–20.5). Pregnancy related anxiety was also found to be a positive predictor of prenatal depression (COR = 1.731; 95% CI: 1.05–2.8, AOR = 2.016; 95% CI: 1.13–3.5). The blood relationship with the husband did not show any significant association with prenatal depression on bivariate analysis and multivariable analysis. Presence of catastrophic events over the past 1 year imposed a two times higher and significant risk of developing prenatal depression among the respondents (COR = 1.969; 95% CI: 1.14–3.37, AOR = 2.148; 95% CI: 1.20–3.83, *p*-value = 0.010). History of mental illness was not included in the analysis because only one respondent had history of this kind and was undergoing treatment with medications.

**Table 4:** Association of maternal depression with social support, marital discord, and domestic violence among the pregnant mothers (*N* = 280).

**Maternal characteristics**	**Depressed mothers (>13) (*n* = 100)**	**Non-depressed mothers (<13) (*n* = 180)**	**Bivariate analysis crude OR (95% CI)**	***P*-value**	**Multivariate analysis adjusted OR (95% CI)**	***P*-value**
**SOCIAL SUPPORT**						
High support (1–2.9)	44 (44.0%)	94 (52.2%)	1		1	
Moderate support (3–5)	24 (24.0%)	54 (30.0%)	0.949 (0.521–1.729)	0.865	0.839 (0.436–1.616)	0.600
Low support (5.1–7)	32 (32.0%)	32 (17.8%)	2.136 (1.164–3.919)	0.014	1.785 (0.915–3.481)	0.089
**MARITAL DISCORD**						
No	32 (32.0%)	79 (43.9%)	1		1	
Yes	68 (68.0%)	101 (56.1%)	1.662 (0.995–2.776)	0.052	1.517 (0.862–2.671)	0.149
**SPOUSE PHYSICAL AND SEXUAL VIOLENCE**						
Yes	11 (11.0%)	04 (2.2%)	5.438 (1.684–17.564)	0.005	5.916 (1.703–20.558)	0.005^*^
No	89 (89.0%)	176 (97.8%)	1		1	
**PREGNANCY RELATED ANXIETY**						
Absent	47 (47.0%)	109 (60.6%)	1		1	
Present	53 (53.0%)	71 (39.4%)	1.731 (1.057–2.836)	0.029	2.016 (1.134–3.587)	0.017^*^
**CONSANGUINITY**						
No	77 (77.0%)	121 (67.2%)	1			
Yes	23 (23.0%)	59 (32.8%)	0.613 (0.350–1.073)	0.086	0.728 (0.397–1.334)	0.304
**CATASTROPHIC EVENTS**						
No	64 (31.4%)	140 (68.6%)	1		1	
Yes	36 (47.4%)	40 (68.6%)	1.969 (1.149–3.374)	0.014	2.148 (1.203–3.837)	0.010^*^

* Positively significant according to multivariable analysis.

### Association of Prenatal Depression With Physiologic Parameters (Table 5)

Table 5 shows the association between physiologic parameters with prenatal depression. Presence of anemia (COR = 1.621; 95% CI: 0.9–2.7, AOR = 1.586; 95% CI: 0.91–2.75) showed some strong association with prenatal depression although this was not statistically significant. No association found between BMI and depression.

**Table 5:** Association of prenatal depression with physiological parameters (*N* = 280).

**Physiological parameters**	**Depressed mothers (>13) (*n* = 100)**	**Non-depressed mothers (<13) (*n* = 180)**	**Bivariate analysis crude OR (95% CI)**	***P*-value**	**Multivariate analysis adjusted OR (95% CI)**	***P*-value**
**BODY MASS INDEX**						
Normal	56 (56.0%)	96 (53.3%)	1			
Underweight	12 (12.0%)	21 (11.7%)	0.980 (0.448–2.141)	0.959		
Obese	32 (32.0%)	63 (35.0%)	0.871 (0.508–1.491)	0.614		
**ANEMIA**						
Present	41 (41.0%)	54 (30.0%)	1.621 (0.973–2.701)	0.063	1.586 (0.912–2.756)	0.102
Absent	59 (59.0%)	126 (70.0%)	1		1	

## Discussion

In this study we have measured the prevalence of prenatal depression among pregnant women and its association with certain risk factors such as socio-demographic characteristics, obstetric history, social support, marital discord, spouse physical and sexual violence, and physiologic measurements which included body mass index and hemoglobin level.

The mean age of respondents was 23.02 ± 3.40 years, which reflects upon the Indian cultural tradition of early marriage and parenthood. The prevalence of depression during pregnancy was 37.8% which is suggestive of a high probability of depression (using an EPDS cutoff score ≥13) among the respondents. The prevalence of prenatal depression makes it a significant public health issue in the study region. EPDS has been validated for use in India and Karnataka (26). Our study used a cut off score of more than or equal to 13 to identify women with depression; this yields a sensitivity of 100% and specificity of 84.9% in Indian settings (27). Another study from Karnataka showed an almost similar prevalence of 36.8% (21) whereas George et al., Ajinkya et al., and Bavle et al. observed much lower prevalence rates of 16.3% in coastal south India (35), 9.18% in Navi Mumbai (22), and 12.3% in Bangalore (36), respectively. This difference could be attributed to diversity in the socio-economic status, socio-cultural and psychosocial factors such as social support which might vary across different regions in the country. Moreover, this study was conducted in a public sector hospital setting, which in itself could pose as a risk factor and predictor for prenatal depression (28) due to inadequate quality of care in such settings.

In our study, majority of the study participants belonged to the low income group. Although we could not document a significant association with socio-economic status, the risk of depression during, and after pregnancy is higher among the socially disadvantaged group (10, 37, 38). It is hypothesized that low income increases the likelihood of poor living conditions, financial struggle and influences interpersonal relationships which could lead to psychosocial stress. Over a third of the study participants were high school graduates though over 90% were not working; however there was no association of education and occupation with depression. Bavle et al. (36) in their study among pregnant women in Bangalore observed that being educated but not employed outside the house could predispose to depression during pregnancy. Study findings from other low income settings point toward a significant association of a woman's occupation with depression: women who were housewives or employed in the private sector or as a laborer or merchant business were prone to get depressed during pregnancy (39, 40). Other socio-demographic factors such as age, husband's education, and occupation did not predict the occurrence of depression in the present study even though some studies have identified young age as a risk factor (41, 42). In Asian settings, having an unemployed or uneducated husband increases the probability of depression (43, 44).

Among the obstetric history variables, unplanned pregnancy increased the odds of depression on bivariate analysis. However, no significant association was observed on multivariate logistic regression analysis. Other studies show that the chance of getting depressed is higher in case of an unplanned pregnancy (15, 37, 45). Similarly multigravidity appeared to be risk factor for depression on bivariate analysis but not on logistic regression analysis although some studies do report a significant relationship (36, 46).

In this study, among the psychosocial factors, presence of spouse physical and sexual violence and pregnancy related anxiety were significant risk factors for prenatal depression in the multivariable analysis. Earlier research has also reported a strong relationship between domestic violence and the risk of depression in pregnancy in high as well as middle to low—income settings (47, 48). Moderate and low social support were significantly related on bivariate but not on multivariate analysis. The linkage between poor social support and prenatal depression has been well-documented (49, 50). Low social support may increase mental stress by inducing feelings of insecurity, predispose toward substance abuse (51), and promote interpersonal conflict (52). The findings from the present study are concurrent with the study results reported by Nongrum et al. India (53), George et al. in Southern India (35), Silva et al. in Brazil (42), and Bernard et al. in Jamaica (54). Depression and anxiety show frequent co-existence and anxiety may emerge as a strong predictor for depression (24, 37). Mohamad et al. (55) and Edward et al. (56) also demonstrated that anxiety strongly increased the risk of suffering from depression during pregnancy. Even a history of mental illness can pose as a risk factor for depression (57, 58); however in our study only one respondent appeared to have such a history. Marital discord appeared predict presence of depression on bivariate but not on multivariate analysis; other studies report that this is e a well-established risk factor due to its influence on social support (59, 60). Likewise consanguinity seemed to be associated with depression only on bivariate testing. Consanguineous marriages are fairly prevalent in South India and clinical observations have reported a high prevalence of depression in such communities (61) which could be genetically driven (62). A major catastrophic event in the past 1 year was an important risk factor which was significantly associated with prenatal depression in this study; this is consistent with the study results reported by Leigh et al. (63) and Shakeel et al. (58). Another study reported that negative life events may lead to persistent higher levels of depressive symptoms since positive life events can decrease the severity of depression over time (64).

Among the physiologic measurements, anemia was significantly associated with depression on bivariate although not so on multivariate analysis. This is in agreement with the study findings reported by Lukose et al. (65); however Yilmaz et al. depicted the existence of such an association between depressive symptoms and anemia in the third trimester of pregnancy (66). Body mass index was not linked with the risk of prenatal depression in the present study. Research done in other countries reportedly point toward an interconnection between obesity and depression (67, 68). The causal pathway could include inflammation (62), hormonal imbalance (69), or sleep disturbance (70).

## Study Strengths And Limitations

This study focuses on prenatal depression which has received less attention than postnatal depression. All the instruments/scales used to measure the study variables had good psychometric properties. Our study had few a limitations. Antenatal care at such hospitals is mostly availed by pregnant women from the lower and middle—income groups in a community. Hence the findings from this study cannot be extrapolated to pregnant women belonging to the high income group as there could be variations in the psychosocial factors and standard of living. As a part of the cohort study protocol, we excluded women with high risk pregnancies and those with a history of intake of steroidal medication over the past 1 year; this could limit the generalizability of the study findings. Adverse obstetric complications during pregnancy can modulate the mental health of a woman during pregnancy. In the south-east Asian context, conflict with in-laws is also a significant risk factor, although this item was not recorded in the present study but will be included in future data collection. We used the EPDS scale which is a self –reporting screening measure for identifying women at risk for depression. Even though EPDS has a high sensitivity and specificity and can be easily administered by a trained health worker, it is important to confirm the presence of depression by using a structured clinical interview to confirm diagnosis.

## Conclusion

The present study showed a high prevalence of prenatal depression which is suggestive of its public health importance in the study region. Spouse physical and sexual violence, pregnancy related anxiety and a history of catastrophic events were important predictors of prenatal depression. Obstetric practice should include screening and diagnosis of prenatal depression as a part of routine antenatal care in low and middle—income countries.

## Ethics Statement

This study was carried out in accordance with the recommendations of the Ethical Committee of Indian Institute of Public Health Bangalore campus (IIPHHB/TRCIEC/118/2017) with written informed consent from all subjects. All subjects gave written informed consent in accordance with the Declaration of Helsinki. The protocol was approved by the Ethical committee of Indian Institute of Public Health Bangalore campus.

## Author Contributions

AN and MK: conceptualization. BS, SV, and JV: formal analysis. AN and GM: funding acquisition. CM: methodology. AN and SB: writing-original draft preparation.

### Conflict of Interest Statement

The authors declare that the research was conducted in the absence of any commercial or financial relationships that could be construed as a potential conflict of interest.
